# Block Magnets with Uniform Core–Shell Microstructure Regenerated from NdFeB Grain Boundary Diffusion Sheet Magnets

**DOI:** 10.3390/nano15181437

**Published:** 2025-09-18

**Authors:** Xiangheng Zhuge, Shuhan Dong, Yuxin Jin, Qiong Wu, Ming Yue, Weiqiang Liu, Yuqing Li, Zhanjia Wang, Qingmei Lu, Yiming Qiu, Yanjie Tong

**Affiliations:** 1 School of Materials Science and Engineering, Beijing University of Technology, Beijing 100124, Chinachangshu@emails.bjut.edu.cn (Y.J.);; 2 State Key Laboratory of Materials Low-Carbon Recycling, Beijing University of Technology, Beijing 100124, China; 3 Physis Motion Control Solution Wuhan Co., Ltd., Wuhan 430000, China

**Keywords:** short-loop process, GBD magnet, regenerated block magnet, coercivity, uniform core–shell microstructure

## Abstract

The grain boundary diffusion (GBD) process is currently the relatively effective method for utilizing heavy rare earth (HRE) elements in NdFeB magnets, especially for magnetic sheets. However, due to a highly uneven microstructure, the recovery of GBD magnets was considered difficult. In this work, our study prioritized short-loop recycling of GBD NdFeB sheet magnets to prepare block magnets. A comparative investigation was conducted between GBD-processed NdFeB magnets and the conventional sintered magnets, with particular emphasis on their recyclability characteristics. Among them, the Tb content of GBD magnets of 0.4 wt.% was significantly lower than sintered magnets of 1.73 wt.%. When two waste magnets were supplemented with the same amount of rare earth, it was found that the coercivity of the block magnets regenerated from GBD sheet magnets was higher. Microstructural analysis revealed that the core–shell grains originally located in the surface layer of GBD magnets were uniformly mixed and diffused with the ordinary particles originally located inside during the regeneration sintering process. The regenerated GBD magnets exhibited a more uniform core–shell microstructure with submicron shells of Tb elements along with reduced areas of RE-rich phase enrichment which facilitated the formation of a continuous and uniform thin-layer grain boundary, thereby enhancing the magnetic isolation effect. Apart from the significance of recycling, these block magnets regenerated from GBD magnets also provides a new approach to solving the challenge of high coercivity and low HRE elements in bulk magnets.

## 1. Introduction

NdFeB permanent magnets have become the dominant material in the rare earth permanent magnet industry [1], holding over 94% of the market share, owing to their exceptional energy transfer efficiency in applications such as renewable energy systems, commercial satellites, autonomous vehicles, and 5G communications [2,3]. Investigations estimate that the global annual quantity of discarded and unrecycled end-of-life magnets may range between 80,000 and 150,000 metric tons [4]. Therefore, regenerating NdFeB waste has acquired considerable strategic significance in building an efficient circular economy for rare earth resources [4,5,6,7]. The recovery of rare earth elements from waste magnets primarily falls into three categories: hydrometallurgical [8,9,10], pyrometallurgical [11,12], and short-loop recycling [13,14,15]. Hydrometallurgical recycling was applicable to the recovery of complex and heterogeneous scrap materials. However, the process was often lengthy, consumed large amounts of chemical reagents, and generated significant wastewater. Pyrometallurgical recycling was suitable for various NdFeB waste materials, featuring a short process, minimal wastewater discharge, and high-value products. The short-loop recycling method was suitable for waste with a known composition and offers advantages in many aspects, such as simple processes, environmental friendliness, and low energy consumption. Li et al. and Shao et al. effectively synthesized recycled magnets through the incorporation of REH_x_ or rare earth alloys into hydrogen-decrepitated powders, ultimately obtaining magnetic properties that exceeded those of the original magnets [16,17].

Expensive HRE elements such as Dy and Tb are commonly used to improve the coercivity of NdFeB magnets [18,19,20,21,22], due to the higher anisotropy field (H_A_) of (Dy/Tb)_2_Fe_14_B than Nd_2_Fe_14_B. To reduce the heavy rare earth (HRE) content necessary for high-coercivity magnet production., the GBD process has been developed in recent years [23,24,25,26,27]. This process generates a core–shell microstructure in the surface layer of NdFeB magnets, consisting of an HRE-enriched shell surrounding an HRE-depleted core, which can optimize coercivity of sheet magnets but has a decreasing effect with increasing thickness [28,29]. Since its proposal in 2003, the GBD process has undergone rapid development, achieving large-scale industrial production with 60,000–80,000 tons of GBD magnets annually. Consequently, this results in generating significant quantities of end-of-life GBD magnets [30]. The scale of waste GBD magnets urgently demands recycling and sustainable utilization.

Due to the scarcity of HRE elements, both hydrometallurgical and pyrometallurgical processes are economically unviable for the recycling of GBD magnets [31,32,33]. The short-loop recycling of GBD magnets was also considered difficult because of a highly uneven microstructure. Compared to traditional sintered NdFeB magnets, the recycling of GBD magnets has received limited research attention. Although a recent study successfully fabricated high-performance regenerated GBD magnets, this achievement required secondary GBD treatment and additional HRE [34,35,36,37]. Therefore, developing efficient recycling technologies for waste GBD NdFeB magnets by adding extra HRE-free elements is of significant research value and practical importance [38].

**Figure 1:**
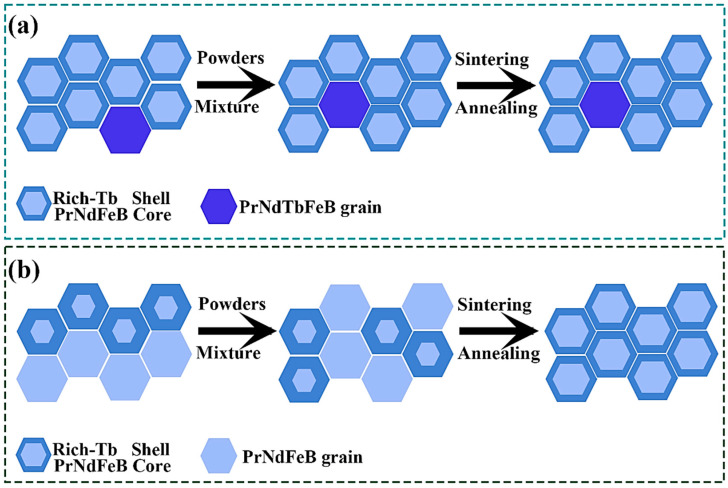
Schematic of Tb element evolution in two types of waste magnets during regeneration process: (**a**) dual-main-phase sintered magnets (Magnet S), (**b**) GBD magnets (Magnet D).

Waste magnets originate from various end-of-life products, primarily including those prepared through the grain boundary diffusion process and the sintering process. In this work, a comparative recycling investigation is presented for GBD magnets and the dual-main-phase sintered magnets. In the regeneration sintering process of dual-main-phase sintered magnets, the elemental distribution has already achieved homogenization during the initial sintering because the distribution characteristics of Tb elements remain essentially unchanged, as shown in Figure 1a. For the recycling of GBD magnets, this work proposes an innovative recycling strategy that synergistically utilizes the inherent core–shell structure powders from waste GBD magnets. As shown in Figure 1b, the core–shell structure powders serve as an additive during the regenerative sintering process, facilitating a more uniform distribution of HRE elements and a thinner shell thickness, which significantly contributes to the coercivity enhancement of block magnets. In this study, Pr_4_Fe_14_B and PrH_x_ were separately added to supplement the total rare earth content, but without adding HRE elements. The coercivity of the block magnets regenerated from GBD sheet magnets were higher compared to regenerated magnets from dual-main-phase sintered magnets with more original HRE. By employing comprehensive microstructural characterization techniques [39], including phase analysis, elemental mapping, and domain structure observation, combined with detailed magnetization reversal studies, the fundamental mechanisms underlying the coercivity enhancement in regenerated magnets with more uniform core–shell microstructures based on waste GBD magnets were elucidated. In conclusion, this study successfully developed an efficient recycling strategy for waste GBD NdFeB magnets, which also provided a new approach to solving the problem of high coercivity and low HRE elements in bulk magnets. This regeneration sintering method based on waste GBD sheets had significant advantages in terms of environmental and economic costs compared to the bulk magnets using specially prepared core–shell structure powders.

## 2. Materials and Methods

Waste GBD magnets (Magnet D) and dual-main-phase sintered magnets (Magnet S) used in this study were disassembled from waste products. The demagnetization treatment was conducted on the waste magnets in a vacuum tube furnace at 400 °C under a vacuum pressure of less than 0.1 Pa for 1 h. And then, the waste magnets were treated in a 0.5 wt.% HNO_3_ solution under ultrasonication to effectively remove the surface phosphating layer. Immediately after, the waste magnets were rinsed sequentially with deionized water and ethanol, followed by a final drying step. The cleaned magnets were subjected to initial size reduction via a jaw crusher operated within an argon environment. Subsequently, the coarse-crushed waste magnets were further refined through HD and jet-milling (JM), yielding single-crystal powders articles with an average size of approximately 4 μm. To compensate for the rare earth element loss in short-loop recycling, PrH_x_ was used as a rare earth supplement for the regenerated magnets. The PrH_x_ powders with X_50_ of approximately 3 μm were obtained using the same HD-JM approach.

**Figure 2:**
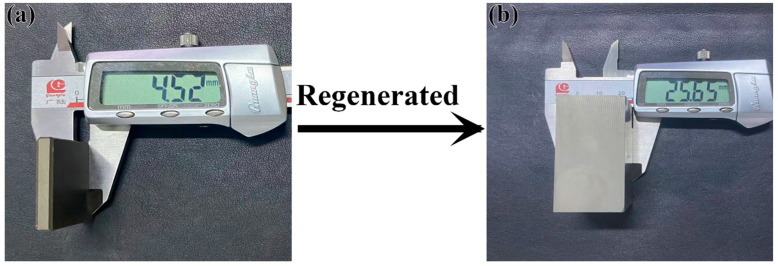
Physical pictures of the magnets: (**a**) waste GBD sheet (Magnet D), (**b**) regenerated magnet (Magnet RD).

The magnet powders and rare earth additives were mixed in specific proportions. Subsequently, the blended powders were aligned within a 1.8 T magnetic field and compressed at 5 MPa, and then cold isostatically pressed at 225 MPa to form green compacts. The compacts were sintered for 3 h at 1060 °C under a vacuum of less than 7 × 10^−3^ Pa, and this was followed by a two-stage annealing treatment at 900 °C for 3 h and 480 °C for 4 h, respectively. Eventually, bulk regenerated sintered magnets were obtained, as shown in Figure 2. In this work, the waste magnets for sintered magnets were denoted as Magnet S, while those from GBD magnets were denoted as Magnet D. A mixture comprising 97.1 wt.% Magnet S and 2.9 wt.% PrH_x_ was denoted as Magnet RS. Similarly, a mixture of 97.1 wt.% Magnet RD and 2.9 wt.% PrH_x_ was denoted as Magnet RD.

The magnetic performance of both original and recycled magnets was precisely characterized employing a NIM-500C closed-loop permanent magnet testing system (National Institute of Metrology, Beijing, China). Compositional analysis was conducted using inductively coupled plasma mass spectrometry (ICP-MS). Phase identification and quantification were performed via X-ray diffraction (XRD) on a Rigaku Ultima IV (Rigaku, Tokyo, Japan) diffractometer (Cu-Kα radiation), followed by Rietveld refinement of the patterns. Density measurements were conducted based on the Archimedes principle, while oxygen and hydrogen concentrations were determined using an OH-3000 gas analyzer. Microstructural characterization was performed using a Hitachi S-3400 N (Hitachi, Tokyo, Japan) scanning electron microscope (SEM) to examine both the original magnets and the sintered/annealed regenerated samples. Elemental distribution analysis was conducted through electron probe microanalysis (EPMA, JEOL JXA-800, Tokyo, Japan) combined with wavelength-dispersive X-ray spectroscopy (WDXS). The evolution of magnetic domain structures during demagnetization was investigated using a magneto-optical Kerr effect microscope (MOKE, BH-786IP-IP-PK, NEOARK, Tokyo, Japan) for all of the regenerated magnet compositions. Detailed measurements of the recoil loops of the two regenerated magnets at 20 °C were made using a vibrating sample magnetometer (VSM, Versa Lab, Quantum Design, San Diego, CA, USA) with a measurement range of −30 kOe to 30 kOe.

## 3. Results and Discussion

**Figure 3:**
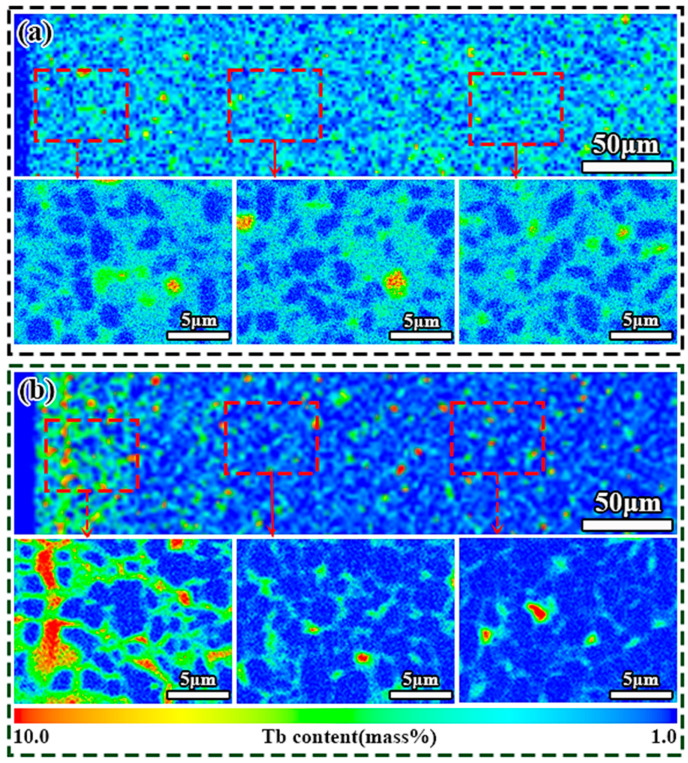
EPMA images of the original magnets: (**a**) Magnet S, (**b**) Magnet D.

Elemental composition and concentration of the waste magnets were characterized in detail. As shown in Table 1, the rare earth content in Magnet S and Magnet D is comparable. Both waste magnets contained Tb as the only HRE element, but Magnet D had a significantly lower content (0.4 wt.%) than Magnet S (1.73 wt.%). Given the significant differences in HRE element content, EPMA elemental analysis was performed on the surface parallel to the c-axis. Figure 3a shows a homogeneous Tb distribution in Magnet S, consistent with the typical elemental distribution of dual-main-phase sintered magnets. In contrast, Magnet D exhibits a Tb concentration gradient, confirming its preparation through the GBD process, as shown in Figure 3b. This finding also explains the difference in the Tb concentration elements between Magnet S and Magnet D, as revealed by ICP results (Table 1). To conserve the use of HRE resources, we adopted PrH_x_ to compensate for the rare earth loss during the short-loop recycling.

**Figure 4:**
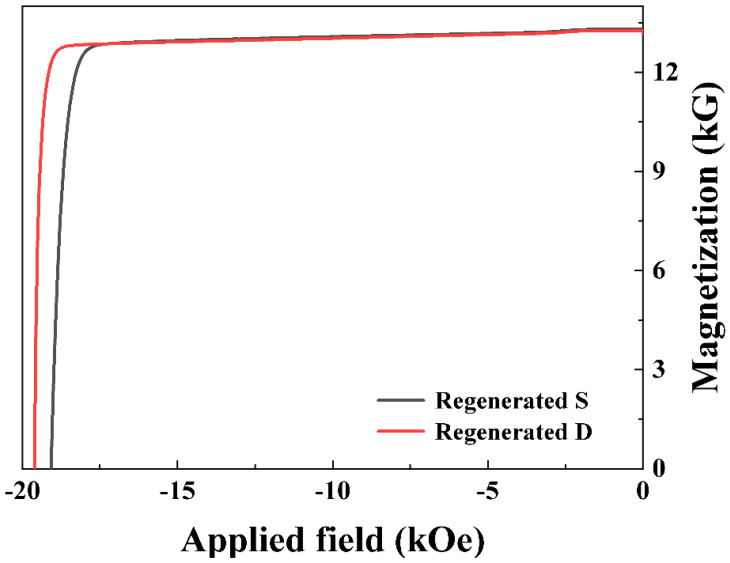
Room temperature demagnetization curves of the regenerated magnets.

Figure 4 illustrates the demagnetization curves of the two regenerated sintered magnets. Meanwhile, Table 2 presents detailed magnetic properties parameters for each of the magnets. Magnet RD displayed a coercivity 0.53 kOe higher and improved squareness compared to Magnet RS. Both samples showed comparable maximum energy products, with Magnet RD exhibiting the least remanence difference. However, the Tb concentration in Magnet RD was much lower than that in Magnet RS (Table 1). A comparative assessment of the magnetic performance and ICP-derived composition of recycled magnets revealed that the HRE elements content in the regenerated magnets based on Magnet D was significantly lower than the regenerated magnets derived from Magnet S, but coercivity of the former was higher. When an equal amount of Pr_4_Fe_14_B was added, the regenerated magnets based on Magnet D exhibited higher coercivity (Figure S1 and Table S1, Supplementary Materials). These results indicate that the short-loop processed Magnet D-based regenerated magnets achieve higher coercivity with lower HRE element content.

**Figure 5:**
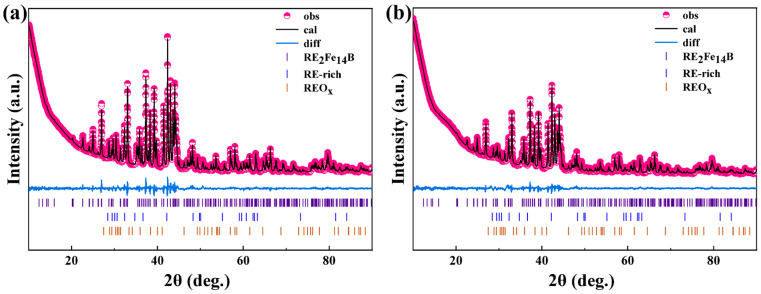
XRD refinement results of the regenerated magnets: (**a**) Magnet RS, (**b**) Magnet RD.

**Figure 6:**
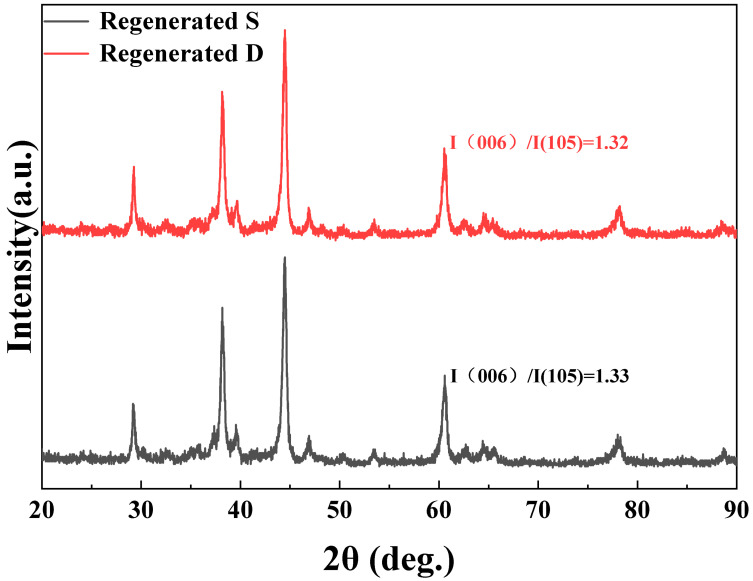
XRD patterns of the regenerated magnets evaluated along the alignment plane.

This study employed the Rietveld refinement method combined with XRD to conduct a detailed analysis of the phase composition of the regenerated magnets, as shown in Figure 5. The phase composition of magnets primarily consists of the RE_2_Fe_14_B phase, with minor amounts of the REO_x_ phase and RE-rich phases, as shown in Table 3. The remanence of NdFeB is predominantly determined by the mass fraction of the main phase RE_2_Fe_14_B, the degree of c-axis texture, and the density. Magnet RS and Magnet RD also demonstrated similar mass fractions of the RE_2_Fe_14_B phase. The degree of texturing is a physical quantity that measures the consistency between the c-axis (easy magnetization axis) of each grain within the magnet and the ideal orientation direction (usually the magnetization direction). A higher orientation degree indicates a more consistent magnetic direction of all grains. The degree of texturing directly determines the maximum magnetic energy product of the magnet and affects its coercivity. XRD patterns collected from the surface normal to the c-axis for both magnets (Figure 6) employed the intensity ratio I _(006)_/I _(105)_ to characterize the degree of texture in NdFeB sintered magnets. The two regenerated magnets exhibited nearly identical I _(006)_/I _(105)_ values, approximately 1.33. The two regenerated magnets exhibited comparable densities, and their densities were approximately 7.5 g/cm^3^. Notably, the oxygen content exhibited a gradient distribution, with the surface layer containing approximately 2700 ppm while the core region showed a lower concentration of approximately 2200 ppm. The above analysis explains why the remanence of the two regenerated magnets was similar. Interestingly, in the two regenerated magnets there was almost no difference in the content of RE-rich and REO_x_ phases. Therefore, the difference in coercivity was almost unaffected by the concentration of the RE-rich phases and REO_x_ phases.

**Figure 7:**
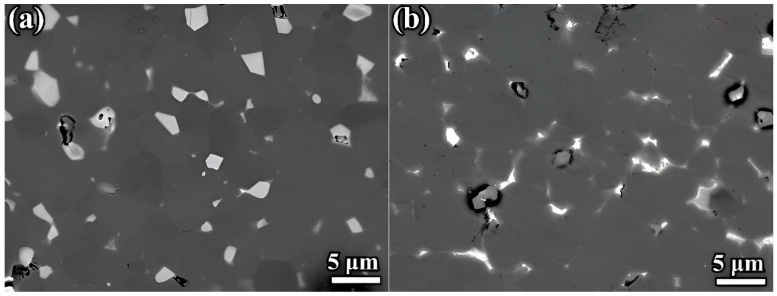
SEM images of the regenerated magnets: (**a**) Magnet RS, (**b**) Magnet RD.

To elucidate the enhanced coercivity in Magnet RD compared to Magnet RS, a detailed analysis of the microstructures of the two regenerated magnets we analyzed was conducted. The microstructure, particularly the distribution of the lamellar grain boundary (GB) phase between the RE_2_Fe_14_B phase grain, plays a critical role in determining the magnetic performance of rare earth permanent magnets, with its specific microstructure significantly contributing to the enhancement of coercivity. As shown in Figure 7, SEM analysis of both recycled magnets reveals a microstructure primarily composed of dark gray RE_2_Fe_14_B phase particles and two distinct types of RE-rich intergranular phases with different contrasts: blocky white RE-rich phases and light gray thin-layer GB phases. A comparative microstructural analysis reveals that Magnet RS developed block RE-rich phases while failing to form distinct thin-layer GB phases, resulting in direct intergranular coupling between adjacent grains. In contrast, Magnet RD formed more continuous and distinct thin-layer GB phases, which effectively enhanced magnetic isolation between adjacent RE_2_Fe_14_B grains, thereby significantly improving coercivity. Similar effects were observed in regenerated magnets prepared with identical Pr_4_Fe_14_B additions to both Magnet S and Magnet D (Figure S2, Supplementary Materials). Above comparative analysis revealed that the regenerated magnets derived from Magnet D with lower HRE element content had significantly higher coercivity than those based on Magnet S with higher HRE element content.

**Figure 8:**
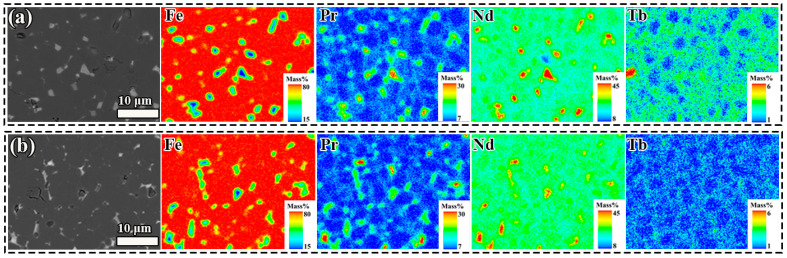
EPMA images of the regenerated magnets: (**a**) Magnet RS, (**b**) Magnet RD.

Elemental distributions of Fe, Pr, Nd, and Tb are analyzed by electron probe microanalysis mapping (EPMA), as shown in Figure 8. The EPMA results reveal distinct distribution regions: Fe was predominantly concentrated in the RE_2_Fe_14_B phase while Pr and Nd exhibited dual distribution in both the RE_2_Fe_14_B phase and GB phase. Notably, the Tb distribution displayed substantial variations between the two types of regenerated magnets. Magnet RS exhibited two types of grains: one forming a Tb-rich distribution and the other consisting of PrNdTbFeB grains. These originated from the two main-phase grains in dual-main-phase magnets. In Magnet RD, a uniform core–shell structured grain formation was observed, with uniformly distributed Tb-rich shells that exhibited relatively lower Tb content and thinner shell thickness. Previous studies indicated that magnet coercivity improves substantially once the HRE element shell attains submicron thickness [24,25]. However, excessive increases in shell thickness or HRE element content do not yield further enhancement in coercivity. ICP results (Table 1) indicated that Magnet RS contained higher Tb concentration (1.50 wt.%) compared to Magnet RD (0.39 wt.%). Although Magnet RD contained less Tb than Magnet RS, Magnet RS showed localized Tb enrichment, but Magnet RD demonstrated homogeneous Tb dispersion across its entire microstructure. Through the short-loop recycling method, the core–shell structure of Magnet D was successfully preserved in Magnet RD, with a more uniform and thinner submicron shell. This structural feature markedly reduced intergranular ferromagnetic coupling among adjacent RE_2_Fe_14_B grains, leading to enhanced coercivity in the recycled magnet. For the two waste magnets doped with an equivalent content of Pr-rich alloy (Pr_4_Fe_14_B), the regenerated magnets based on Magnet D also formed a more uniform and homogeneous distribution of Tb elements (Figure S3, Supplementary Materials). This enhanced demagnetization coupling effects, resulting in improved coercivity.

**Figure 9:**
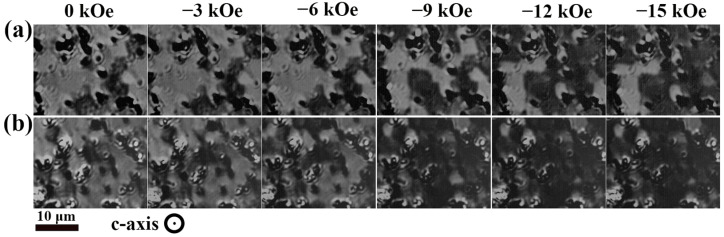
Domain evolution of the regenerated magnets during the magnetization reversal process: (**a**) Magnet RS, (**b**) Magnet RD.

Figure 9 illustrates the domain evolution during magnetization reversal in both recycled magnets. Prior to imaging, the samples were saturated along the c-axis using a 100 kOe pulsed magnetic field. To observe the magnetization reversal in both recycled magnets, a magnetic field normal to the observation plane was gradually increased from 0 to −15 kOe. Owing to demagnetization effects, multiple multidomain structures formed at remanence (0 kOe), with distinct bright-contrast positive domains also observed. Combined with the EPMA elemental analysis results in Figure 6, the differences in Tb distribution within the regenerated magnets caused substantial variations in the magnetization reversal process. With increasing reverse field, the positive domains in Magnet RS reversed asynchronously, mainly due to the higher Tb content and its non-uniform distribution in the recycled magnets. Specifically, regions with higher Tb concentration demonstrated a significantly larger anisotropy field, thereby inhibiting magnetization reversal at the last magnetic field (−15 kOe). In contrast, regions with lower Tb concentrations were easier to reverse due to their reduced anisotropy field. A small number of regions with elevated Tb concentration did not contribute to the coercivity. Therefore, in a sense these HRE elements have been wasted. In Magnet RD, when the applied reverse field was increased to −6 kOe, the reversal of positive domain regions exhibited minimal changes. Upon further increasing the reverse field to −9 kOe, a significant portion of the positive domain regions-initiated reversal. The combined analysis of EPMA and magnetic domain observations revealed that the uniform Tb distribution in regenerated magnets promoted synchronous reversal of positive domains under an increasing reversal field. Likewise, when both waste magnets were doped with Pr_4_Fe_14_B the regenerated magnet based on Magnet D also exhibited uniform magnetization reversal behavior (Figure S4, Supplementary Materials). The observed structural evolution demonstrated that the more homogeneous Tb distribution in the regenerated magnets derived from GBD magnets results in more consistent magnetization reversal behavior, which contributed to enhancing the coercivity of the regenerated magnet.

**Figure 10:**
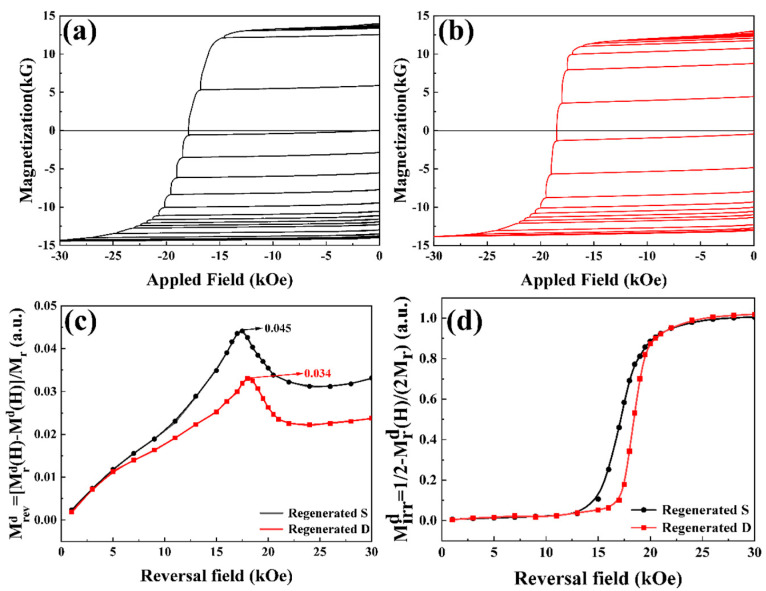
Recoil loops of the regenerated magnets. (**a**) Magnet RS. (**b**) Magnet RD. (**c**) Reversible portion dependence of the reverse magnetic field. (**d**) Irreversible portion dependence of the reverse magnetic field.

To gain deeper insights into the magnetization reversal mechanisms of the regenerated sintered magnets, we conducted systematic measurements of their magnetic recoil loops. Figure 10a,b displays the recoil loops of the regenerated magnets during the magnetization reversal process, illustrating the reversible and irreversible portion dependence as functions of the applied reverse magnetic field. The reversible $M_{rev}^{d}\left(H\right)=\left[M_{r}^{d}\left(H\right)-M^{d}\left(H\right)\right]/M_{r}$ and the irreversible $M_{irr}^{d}\left(H\right)=\frac{1}{2}-M_{r}^{d}(H)/(2M_{r})$ portion dependence of the reversal field were obtained by the recoil loops. The reversible and irreversible magnetization reversal components indicated the distribution of intergranular ferromagnetic coupling strength and effective anisotropy. As shown in Figure 10c, the maximum reversible portion in Magnet RD decreases to 3.4% of its remanence, whereas that value reaches 4.5% in Magnet RS. This suggests that the ferromagnetic coupling in Magnet RD was substantially weakened, which resulted from the development of continuous lamellar grain boundaries with low Fe concentration between most RE_2_Fe_14_B grains. Figure 10d shows the relationship of the irreversible portion as a function of the reversal field. With increasing reversal field, the irreversible magnetization component rises progressively in both recycled magnets. Upon reaching the irreversible nucleation field (H_n_), the irreversible component increased abruptly due to the onset of grain reversal. The maximum irreversible nucleation field of Magnet RD was greater than that of Magnet RS, indicating that Magnet RD experienced greater resistance to nucleation and domain expansion. Therefore, Magnet RD demonstrated more uniform reversal regions in the remanence state and exhibited the strongest resistance to the reversal fields during the magnetization reversal process. When equivalent amounts of Pr_4_Fe_14_B were added to both Magnet S and Magnet D, the regenerated magnet based on Magnet D exhibited a smaller reversible portion and larger maximum reversible nucleation field (Figure S5, Supplementary Materials). The above shows that the regenerated magnets derived from Magnet D have a better magnetization reversal mechanism. Finally, the recycled magnets exhibited a Vickers hardness of approximately 570 kg/mm^2^, a three-point flexural strength of about 396 MPa, and a density of 7.48 g/cm^3^. The results show that the mechanical properties of the recycled magnets are slightly lower than those of the original magnets, but they still remain within the applicable range.

## 4. Conclusions

This study employed the short-loop process to recycle GBD NdFeB sheet magnets extracted from waste products, utilizing PrH_x_ and Pr_4_Fe_14_B to enhance the magnetic performance of regenerated NdFeB. Notably, when doped with the same content of non-HRE element additives, the regenerated magnets based on GBD sheet magnets with lower HRE element content exhibited higher coercivity compared to those obtained from the recovery of dual-main-phase sintered magnets.

Microscopic analysis revealed that the regenerated Magnet D exhibited a more distinct and continuous GB phase, along with smaller and fewer RE-rich phase aggregation areas. The core–shell structure of the waste Magnet D was partially preserved during the short-loop regeneration, leading to a more dispersed and uniform distribution of Tb elements in the regenerated magnet. This preserved weak core–shell structure with submicron shells of Tb elements facilitated enhanced magnetic isolation between RE_2_Fe_14_B grains, which contributed to the improved coercivity.

The aforementioned findings offer significant technical insights into the recycling of GBD magnets, providing a reliable reference for the sustainable recovery of rare earth resources.

## Figures and Tables

**Table 1:** Elemental compositions of waste magnets and regenerated magnets.

Element (wt%)	RE	Nd	Pr	Tb	B	Fe	Co	Cu	Al	Zr	Ti	Ga
Magnet S	31.41	22.36	7.32	1.73	0.93	65.74	1.25	0.11	0.25	0.11	ND	0.20
Magnet D	31.20	23.64	7.16	0.40	0.94	66.73	0.53	0.07	0.26	0.10	0.09	0.08
Magnet RS	31.51	20.57	9.44	1.50	0.92	65.73	1.20	0.11	0.23	0.11	ND	0.19
Magnet RD	31.33	21.66	9.28	0.39	0.92	66.66	0.51	0.08	0.15	0.10	0.09	0.06

**Table 2:** The magnetic performance parameters of the regenerated magnets.

Magnet	B_r_ (kG)	H_cj_ (kOe)	(BH)_max_ (MGOe)	H_k_/H_cj_ (%)
Magnet RS	13.31	19.08	43.21	95.8
Magnet RD	13.27	19.61	42.95	97.7

**Table 3:** Mass fraction of different phases according to the Rietveld analysis and R factors of the regenerated magnets.

Sample	Mass Fraction (wt.%)			R Factor	
	RE_2_Fe_14_B	RE-rich	REO_x_	R_p_	R_wp_
Magnet RS	96.88	2.36	0.76	1.76	2.63
Magnet RD	96.95	2.28	0.77	1.52	2.20

## Supplementary Materials

The following supporting information can be downloaded at: https://www.mdpi.com/article/10.3390/nano15181437/s1, Figure S1: Room-temperature demagnetization curves of the regenerated magnets; Table S1: The magnetic performance parameters of the regenerated magnets; Figure S2: SEM images of the regenerated magnets: (a) Magnet S+Pr_4_Fe_14_B, (b) Magnet D+Pr_4_Fe_14_B; Figure S3: EPMA images of the regenerated magnets: (a) Magnet S+Pr_4_Fe_14_B; Figure S4: Domain evolution of the regenerated magnets during the magnetization reversal process: (a) Magnet S+Pr_4_Fe_14_B, (b) Magnet D+Pr_4_Fe_14_B; Figure S5: Recoil loops of the regenerated magnets: (a) Magnet S+Pr_4_Fe_14_B, (b) Magnet D+Pr_4_Fe_14_B; (c) Reversible portions dependence of the reverse magnetic field; (d) Irreversible portions dependence of the reverse magnetic field.
