# Supplementary material for: Block Magnets with Uniform Core–Shell Microstructure Regenerated from NdFeB Grain Boundary Diffusion Sheet Magnets
Source: Nanomaterials (Basel). 2025 Sep 18;15(18):1437. doi: 10.3390/nano15181437 (PMC12472906; doi:10.3390/nano15181437)
Supplement: Supplementary file 1 [file nanomaterials-15-01437-s001.zip › nanomaterials-3835533-supplementary.pdf]

Article

Block Magnets with Uniform Core–Shell Microstructure Regenerated from NdFeB Grain Boundary Diffusion Sheet Magnets

Xiangheng Zhuge [1], Shuhan Dong [1], Yuxin Jin [1], Qiong Wu [1,2,*], Ming Yue [1,2,*], Weiqiang Liu [1,2], Yuqing Li [1,2], Zhanjia Wang [1,2], Qingmei Lu [1,2], Yiming Qiu [3] and Yanjie Tong [3]

[1] School of Materials Science and Engineering, Beijing University of Technology, Beijing 100124, China; cst1020@emails.bjut.edu.cn (X.Z.); dongshuhan@emails.bjut.edu.cn (S.D.); changshu@emails.bjut.edu.cn (Y.J.); liuwq@bjut.edu.cn (W.L.); yqli@bjut.edu.cn (Y.L.); wangzhanjia@bjut.edu.cn (Z.W.); qmlu@bjut.edu.cn (Q.L.)
[2] State Key Laboratory of Materials Low-Carbon Recycling, Beijing University of Technology, Beijing 100124, China
[3] Physis Motion Control Solution Wuhan Co., Ltd., Wuhan 430000, China; qiuyiming@physis.com.cn (Y.Q.); tongyanjie@physis.com.cn (Y.T.)
* Correspondence: wuqiong0506@bjut.edu.cn (Q.W.); yueming@bjut.edu.cn (M.Y.)

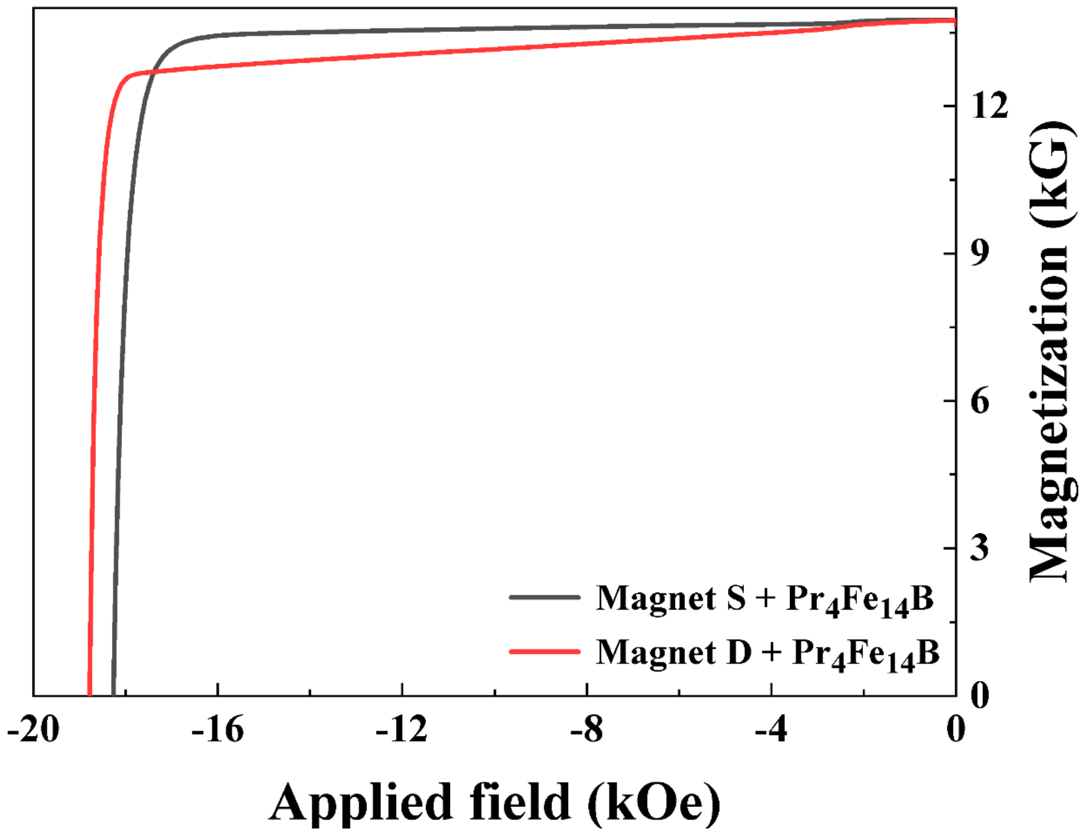

Figure S1. Room-temperature demagnetization curves of the regenerated magnets.

Table S1. The magnetic performance parameters of the regenerated magnets.

Magnet	B_r (kG)	H_{cj} (kOe)	$(BH)_{max}$ (MGOe)	H_k/H_{cj} (%)
Magnet S+$Pr_4Fe_{14}B$	13.74	18.26	46.49	95.8
Magnet D+$Pr_4Fe_{14}B$	13.76	18.85	44.69	96.8

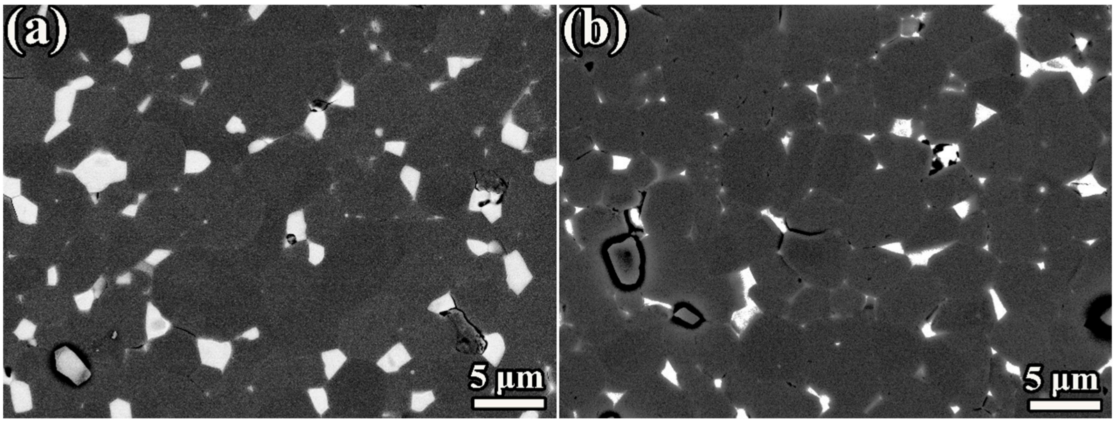

Figure S2. SEM images of the regenerated magnets: (a) Magnet S+$Pr_4Fe_{14}B$, (b) Magnet D+$Pr_4Fe_{14}B$.

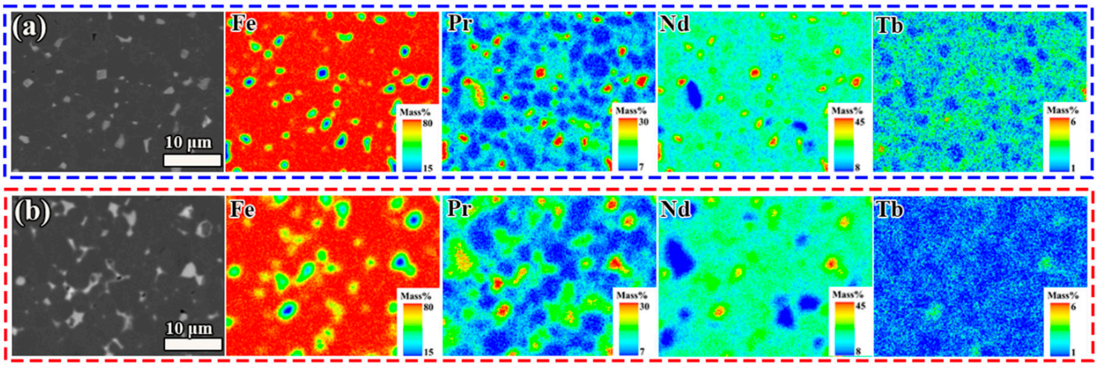

Figure S3. EPMA images of the regenerated magnets: (a) Magnet S+$Pr_4Fe_{14}B$, (b) Magnet D+$Pr_4Fe_{14}B$.

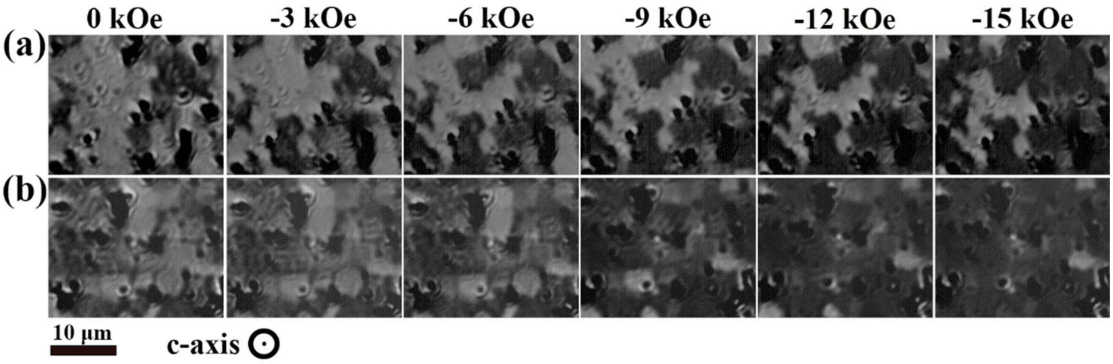

Figure S4. Domain evolution of the regenerated magnets during the magnetization reversal process: (a) Magnet S+$Pr_4Fe_{14}B$, (b) Magnet D+$Pr_4Fe_{14}B$.

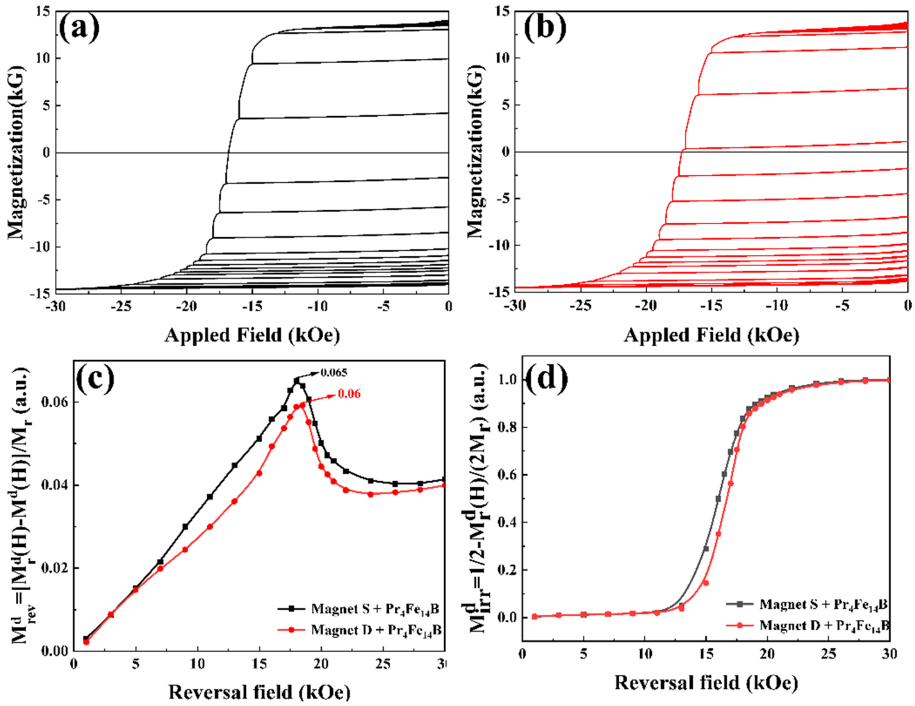

Figure S5. Recoil loops of the regenerated magnets: (a) Magnet S+$Pr_4Fe_{14}B$, (b) Magnet D+$Pr_4Fe_{14}B$; (c) Reversible portions dependence of the reverse magnetic field; (d) Irreversible portions dependence of the reverse magnetic field.
